# Hospital Admissions for Hypertensive Crisis in the Emergency Departments: A Large Multicenter Italian Study

**DOI:** 10.1371/journal.pone.0093542

**Published:** 2014-04-02

**Authors:** Giuliano Pinna, Claudio Pascale, Paolo Fornengo, Sebastiana Arras, Carmela Piras, Pietro Panzarasa, Gianpaolo Carmosino, Orietta Franza, Vincenzo Semeraro, Salvatore Lenti, Susanna Pietrelli, Sergio Panzone, Christian Bracco, Roberto Fiorini, Giovanni Rastelli, Daniela Bergandi, Bruno Zampaglione, Roberto Musso, Claudio Marengo, Giancarlo Santoro, Sergio Zamboni, Barbara Traversa, Maddalena Barattini, Graziella Bruno

**Affiliations:** 1 Department of Internal Medicine, Ospedale Cottolengo, Torino, Italy; 2 Emergency Department, Ospedale Civile, Alghero, Sassari, Italy; 3 Emergency Department, Ospedale Umberto Parini, Aosta, Italy; 4 Emergency Department, Ospedale Cardinal Massaia, Asti, Italy; 5 Emergency Department, Ospedale San Donato, Arezzo, Italy; 6 Emergency Department, Azienda Ospedaliera Santa Croce e Carle, Cuneo, Italy; 7 Emergency Department, Presidio Ospedaliero, Parma, Italy; 8 Emergency Department, Presidio Ospedale Martini, Torino, Italy; 9 Emergency Department, Ospedale Santa Croce, Moncalieri, Italy, Torino, Italy; 10 Emergency Department, Ospedale Civile, Rovigo, Italy; 11 Emergency Department, Ospedale SS. Antonio e Margherita, Tortona, Italy; 12 Department of Medical Sciences, Univerity of Torino, Torino, Italy; University of British Columbia, Canada

## Abstract

Epidemiological data on the impact of hypertensive crises (emergencies and urgencies) on referral to the Emergency Departments (EDs) are lacking, in spite of the evidence that they may be life-threatening conditions. We performed a multicenter study to identify all patients aged 18 years and over who were admitted to 10 Italian EDs during 2009 for hypertensive crises (systolic blood pressure ≥220 mmHg and/or diastolic blood pressure ≥120 mmHg). We classified patients as affected by either hypertensive emergencies or hypertensive urgencies depending on the presence or the absence of progressive target organ damage, respectively. Logistic regression analysis was then performed to assess variables independently associated with hypertensive emergencies with respect to hypertensive urgencies. Of 333,407 patients admitted to the EDs over the one-year period, 1,546 had hypertensive crises (4.6/1,000, 95% CI 4.4–4.9), and 23% of them had unknown hypertension. Hypertensive emergencies (n = 391, 25.3% of hypertensive crises) were acute pulmonary edema (30.9%), stroke (22.0%,), myocardial infarction (17.9%), acute aortic dissection (7.9%), acute renal failure (5.9%) and hypertensive encephalopathy (4.9%). Men had higher frequency than women of unknown hypertension (27.9% vs 18.5%, p<0.001). Even among known hypertensive patients, a larger proportion of men than women reported not taking anti-hypertensive drug (12.6% among men and 9.4% among women (p<0.001). Compared to women of similar age, men had higher likelihood of having hypertensive emergencies than urgencies (OR = 1.34, 95% CI 1.06–1.70), independently of presenting symptoms, creatinine, smoking habit and known hypertension. This study shows that hypertensive crises involved almost 5 out of 1,000 patients-year admitted to EDs. Sex differences in frequencies of unknown hypertension, compliance to treatment and risk of hypertensive emergencies might have implications for public health programs.

## Introduction

Hypertension is a well-established cardiovascular risk factor, which involves more than 1 billion of persons worldwide [1]–[2]. Chronic hypertension treatment strategies are well codified by international guidelines [3]–[4], whereas few evidence-based recommendations are available on acute severe elevation in blood pressure [2], [5]. Hypertensive crises are defined as hypertensive emergencies and hypertensive urgencies, depending on either presence or absence of acute end-organ dysfunction, respectively [6]–[8]. This classification is relevant from a clinical point of view, as correct diagnosis and appropriate treatment are critical in limiting morbidity and mortality of hypertensive patients [1], [8]–[10]. Indeed, blood pressure should be reduced within 24 to 48 hours in patients with hypertensive urgencies, whereas target values should be obtained over a period of minutes to hours in those with hypertensive emergencies [6], [11], [12]. Moreover, compliance of patients to antihypertensive treatment is likely to affect the risk of hypertensive emergencies, but data on this issue are lacking.

Epidemiological data on prevalence and clinical features of patients referred to the emergency departments (EDs) for hypertensive crises are limited, in spite of their relevance from a public health perspective [13]–[15]. Main limitations of available studies are the recruitment of cases from a single hospital, thus containing the external validity of data, and the low numbers of examined patients, thus restricting the power of results.

Therefore, we performed a multicenter study, including 10 representative EDs distributed throughout Italy, to assess the impact on National Health System of hypertensive crises referred to hospitals during a one-year period and to describe clinical features of patients with hypertensive emergencies and urgencies.

## Materials and Methods

This multicenter study included 10 Italian hospitals distributed throughout the country (6 hospitals in North-West Italy, 1 hospital in North-East Italy, 2 hospitals in Central Italy and one in Sardinia). The study was approved in 2008 by the Interhospital Ethical Committee of the Piedmont Region Italy (Ospedale Cardinal Massaia, Asti; Azienda Ospedaliera Santa Croce e Carle, Cuneo; Presidio Ospedale Martini, Torino; Ospedale SS. Antonio e Margherita, Tortona; Ospedale Santa Croce, Moncalieri), as well as by the local ethic committees of the participating centers (Ospedale Civile, Alghero; Ospedale Umberto Parini, Aosta; Ospedale san Donato, Arezzo; Presidio Ospedaliero, Parma; Ospedale Civile, Rovigo). Accordingly, written/oral informed consent was obtained by either the patient or authorized relatives of patients with severe neurological impairment, and documented by clinical chart. The oral consent was obtained when the neurological condition made impossible to the patient to sign, fulfilling a specific form signed thereafter by the physician and by the authorized relative. This procedure was approved by the ethics committees. The study was conducted in accordance with the Helsinki Declaration. The Italian National Health Service covers all Italian citizens and foreigners, who have free access to EDs either directly or referred by their general practitioners. As there are no private EDs in the Italian Regions involved in the study, recruitment was unbiased by socioeconomic conditions.

We recruited all consecutive persons aged 18 years and over who were admitted to the EDs of participating hospitals in period 01/01/2009-12/31/2009 for hypertensive crisis, defined as systolic blood pressure≥220 mmHg and/or diastolic blood pressure ≥120 mmHg, after unrelated acute problems, such as pain and anxiety, were alleviated. The blood pressure cut-off values were consistent with those adopted by previous studies, to allow data comparisons among studies [13]–[15]. Patients were interviewed and examined by trained investigators, according to standardized measurements and procedures, and those with a previous diagnosis of hypertension were identified through the examination of medical history and current treatments. Blood pressure was measured with the patient in the recumbent position by use of a mercury sphygmomanometer, according to a standard technique, after unrelated acute problems, such as pain and anxiety were alleviated. The average of the second and the third of three consecutive readings taken 1 minute apart was used for analyses. As one recruiting center modified methods of data collection, thus potentially affecting data comparability among centers, it was not included in present analyses, which were finally based on 9 EDs. Women affected by eclampsia and pre-eclampsia were not included also, as they are generally referred to the Obstetrics Clinics without passing through the EDs. Patients with hypertensive crisis were further classified as having either *hypertensive emergencies* or *urgencies* on the basis of presence or absence, respectively, of acute and progressive end-organ damage, such as hypertensive encephalopathy, stroke (either ischemic or due to intracerebral/subarachnoid haemorrhage), acute pulmonary edema, acute aortic dissection, acute myocardial infarction/unstable angina pectoris, progressive renal failure. These conditions were diagnosed on the basis of clinical data and diagnostic tests when appropriate, such as blood and urine chemistry, eye fundus examination, ECG, chest X-ray, computed tomography and ultrasound imaging. Each patient underwent a complete clinical history, physical examination and routine blood and urine chemical analyses. The entity of renal damage was assessed through plasma creatinine values at admission, doubling of creatinine values during clinical monitoring in the EDs and estimated Glomerular Filtration Rate (eGFR), using the four-component abbreviated equation from the MDRD study [16]. Plasma creatinine values were determined using the modified Jaffè method (Creatinine Liquid, Sentinel diagnostics CH SpA, Milan, Italy).

The diagnosis of hypertensive emergency was standardized through a shared protocol. Acute aortic dissection was considered in any patient complaining of chest pain, back pain or abdominal pain associated with high values of blood pressure, and diagnosis confirmed by computed tomography angiography. Acute coronary syndrome included the ST-elevation myocardial infarction (STEMI, ST-segment elevations of more than 0.1 mV in two corresponding leads and a typical rise and fall of cardiac enzymes), the non-ST elevation myocardial infarction (NSTEMI, considered as electrocardiographic ST-segment depression or prominent T-wave inversion and/or positive biomarkers of necrosis in the absence of ST-segment elevation and in an appropriate clinical setting, such as chest discomfort or angina equivalent) and unstable angina pectoris (ischemic symptoms suggestive of an acute coronary syndrome and no elevation in troponin or creatin-kinase-MB, with or without ECG changes indicative of ischemia) with the need of coronary angiography and/or intervention. Acute pulmonary edema was defined as evidence of clinical signs and confirmed by chest radiograph. Systolic and/or diastolic impaired ventricular function was assessed by a standard echocardiography methodology. Hypertensive encephalopathy was defined as progressively appearance of severe headache, nausea, vomiting and visual disorders, with or without localized or generalized seizures. Acute stroke was defined by neurological symptoms (aphasia, hemianopsia, paresthesia or paresis) lasting more than 24 hours and by computer tomography of the brain revealing either ischemic or hemorrhagic area.

Frequencies have been calculated using as numerators the number of hypertensive crises occurring over the study period and as denominators the total number of admissions to the EDs. Differences in clinical characteristics of patients were assessed using the χ^2^ test for categorical variables and the t test for continuous variables. Results are shown as mean ± standard deviation (SD), geometric means and interquartile range (IQR) for non-normally distributed values and frequencies for categorical variables. All reported P values are two-sided and a P value of less than 0.05 was considered to indicate statistical significance. We performed logistic regression analysis to assess the role of age, sex, presenting symptoms (typical vs atypical), current smoke (yes vs no), previously known hypertension (yes vs no), creatinine values (log values) on the risk of having hypertensive emergency with respect to hypertensive urgency. All analyses were performed with STATA, Release 11.0.

## Results

Out of 333,407 patients consecutively admitted to the EDs of recruiting hospitals during the study period, 1,546 had a hypertensive crisis (4.6/1000, 95% CI 4.4–4.9), and 391 of them (25.3%) had hypertensive emergencies. Twenty-three per cent of hypertensive crises occurred in patients with unknown hypertension (27.9% among men and 18.5% among women, p<0.001). Among patients with known hypertension, 12.6% of men and 9.4% of women (p<0.001) referred that they were not taking antihypertensive drugs. As shown in table 1, women were older than men and had statistically significant higher values of both systolic and diastolic blood pressure. After adjustment for age, however, mean adjusted values were similar between genders: systolic blood pressure, 202.5 (95% CI 200.6–204.5) mmHg in men and 205.2 (95% CI 203.4–207.1) mmHg in women; diastolic blood pressure 115.8 (95% CI 114.6–116.9) mmHg in men, and 114.6 (95% CI 113.5–115.8) mmHg in women.

**Table 1:** Characteristics of patients with hypertensive crises recruited in the multicenter Italian study.

	All cohort (n = 1546)	Men (n = 748)	Women (n = 798)	P value
**Age (years)**	69.0±14.1	66.5±14.5	71.4±14.9	<0.0001
**Systolic blood pressure (mmHg)**	203.9±27.4	201.7±28.7	206.0±26.0	0.002
**Diastolic blood pressure (mmHg)**	115.2±16.4	116.3±16.6	114.1±16.2	0.007
**Plasma creatinine value (mg/dl)**	1.09 (1.0–1.3)	1.1 (1.1–1.3)	1.108 (1.0–1.2)	0.17
**Current smokers**	277 (18.0%)	180 (24.0%)	97 (13%)	0.02
**Known hypertension**	1117 (77.0%)	501 (72.1%)	616 (81.5%)	<0.0001
**Patients taking no antihypertensive drug**	118 (10.6%)	63 (12.6%)	58 (9.4%)	0.09
**Treatment for hypertension**				0.010
** None**	280 (25.1%)	144 (28.8%)	136 (21.6%)	
** RAS-inhibitors**	169 (15.1%)	61 (12.2%)	108 (17.8%)	
** Beta-blockers**	48 (4.3%)	18 (3.7%)	30 (4.8%)	
** Calcium-antagonists**	32 (2.9%)	15 (3.0%)	17 (2.8%)	
** Diuretics**	26 (2.3%)	8 (1.7%)	18 (2.8%)	
** Alpha-blockers**	11 (1.1%)	6 (1.2%)	5 (1.1%)	
** Two or more drugs**	551 (49.3%)	248 (49.5%)	303 (49.2%)	

Data are either means ± standard deviation or geometric means and interquartile range.

Among patients taking one antihypertensive drug only, renin angiotensins system (RAS) inhibitors were the most frequently employed drug (Table 1). Patients treated with two or more antihypertensive drugs were 49.3%. As regards ethnic groups, 97% of patients were Europeans, 1.5% were Africans and 1.5% were Asians.

Patients referred to the EDs by their general practitioners were 7.3% and those referred by Territorial Emergency Services were 39.2%. Patients who were not referred were 53.4%, with a statistically higher frequency among those with non-specific symptoms than among those with specific symptoms, (59.4% vs 45.7%p<0.0001). Presenting symptoms were non-specific in 55.6% of patients (Table 2), whereas chest pain and focal neurologic deficits were evident in 28.3% and 16.1% of patients, respectively, with no sex differences.

**Table 2:** Presenting symptoms in patients with hypertensive crises admitted to the EDs in the Italian multicenter study.

Symptoms	Hypertensive crises (n = 1546)	Hypertensive emergencies (n = 391)	Hypertensive urgencies (n = 1155)
**Cardiological symptoms (shortness of breath/chest pain/arrhythmias/syncope)**	437 (28.3%)	109 (28.1%)	328 (28.4%)
**Focal neurologic deficit**	249 (16.1%)	88 (22.6%)	161 (13.9%)
**Non-specific symptoms (headache without neurological deficit/dizziness/epistaxis/vomits/palpitations, etc.)**	860 (55.6%)	192 (49.3%)	666 (57.7%)

Among 391 patients with hypertensive emergencies, 121 (30.9) had acute pulmonary edema, 86 (22.0%) had stroke, including 60 ischemic strokes and 26 hemorrhagic strokes, 70 (17.9%) had myocardial infarction, 31 (7.9%) had acute aortic dissection, 23 (5.9%) had acute renal failure and 19 (4.9%) had hypertensive encephalopathy. Two patients only had both acute pulmonary edema and stroke. With respect to patients with hypertensive urgencies (Table 3), those with hypertensive emergencies were more likely to be men, whereas no significant differences were found in age and in frequencies of unknown hypertension. Statistically significant differences in symptoms at presentation were found between hypertensive urgencies and emergencies, with higher frequency of focal neurological symptoms and lower frequency of non-specific symptoms in the latter (table 3). Among the subgroup of patients with hypertensive emergencies, statistically significant differences were evident comparing patients with known hypertension and those with unknown hypertension. Indeed, the latter had higher frequency of men (51.7% vs 48.3%, p = 0.003), lower age (63.2±1.9 vs 67.5±1.9, p<0.001) and higher frequency of non-specific symptoms (63.5% vs 47.9%, p = 0.036).

**Table 3:** Clinical feature of patients with hypertensive urgencies and emergencies. Data are means ± standard deviation.

	Hypertensive emergencies (n = 391)	Hypertensive urgencies (n = 1155)	P value
Age	69.9±14.3	68.8±15.1	0.20
**Men**	208 (53.2%)	540 (46.8%)	0.03
**Systolic blood pressure (mmHg)**	203.5±29.1	204.0±26.9	0.76
**Diastolic blood pressure (mmHg)**	114.9±17.5	115.3±16.0	0.34
**Plasma creatinine value (mg/dl)**	1.20 (1.00–1.30)	1.16 (1.00–1.20)	0.72
**Current smokers**	78 (20%)	208 (18%)	0.74
**Known hypertension**	309 (79.1%)	881 (76.3%)	0.26
**Patient taking no antihypertensive drug**	28 (9.1%)	98 (11.1%)	0.34
**Current treatment for hypertension**			
**None**	89 (22.9%)	298 (25.8%)	
**RAS-inhibitors**	54 (13.8%)	180 (15.6%)	
**Beta-blockers**	15 (3.8%)	51 (4.4%)	
**Calcium-antagonists**	11 (2.8%)	33 (2.9%)	
**Diuretics**	6 (1.6%)	29 (2.5%)	
**Alpha-blockers**	6 (1.6%)	12 (1.0%)	
**Two or more drugs**	210 (53.6%)	552 (47.8%)	

In logistic regression analyses, after adjustment for age, patients with hypertensive emergencies had higher risk of being men (OR = 1.34, 95% CI 1.06–1.70) and lower likelihood of having non-specific symptoms (OR = 0.72, 95% CI 0.57–0.90) than patients with hypertensive urgencies. Further inclusion in the model of known hypertension, current smoking habit and creatinine values did not contribute significantly to the model.

Table 4 compares the results of epidemiological studies examining frequencies and clinical features of patients with hypertensive crises among those admitted to the EDs.

**Table 4:** Comparison among studies based upon the recruitment of patients with hypertensive crises admitted to EDs.

	Present work	Zampaglione B et al, 1996 [14]	Vilela-Martin et al, 2004 [13]	Vilela-Martin et al, 2010 [15]
**Recruiting centers**	9	1	1	1
**Admissions to EDs (n)**	333.407	14.209	76.723	79.463
**Inclusion criteria**				
Systolic blood pressure (mmHg)	>220	>220	-	-
Diastolic blood pressure (mmHg)	>120	>120	>120	>120
**Hypertensive crises, n (%)**	1546 (0.46)	449 (3.16)	452 (0.50)	362 (0.45)
Age (years)	69.0±14.1	64.0+15.0	53.7±17.8	61.1±14.5
Unknown hypertension (%)	23	23	18.2	11.9
**Hypertensive urgencies, n (%)**	1155 (74.7)	341 (76.0)	273 (60.4)	131 (36.2)
Age (years)	68.8±15.1	60.0±14.0	49.9±18.6	57.0±15.6
Unknown hypertension (%)	23.7	28	19.8	7.7
**Hypertensive emergencies, n (%)**	391 (25.3)	108 (24)	179 (39.6)	231 (63.8)
Age (years)	69.9±14.3	67.0±16.0	59.6±14.8	63.4±13.4
Unknown hypertension (%)	20.9	8.0	15.6	14.3

Data are means ± SD.

## Discussion

This large Italian multicenter study provides evidence that hypertensive crises involved almost 5 out of 1,000 patients admitted over one year to the EDs. Second, hypertensive emergencies represents one fourth of patients admitted for hypertensive crises, with a 34% higher risk in men than in women of similar age. Third, the frequency of patients with unknown hypertension was high, both in patients with hypertensive emergencies and in those with hypertensive urgencies. Finally, the frequency of patients with a previous diagnosis of hypertension who reported that they were taking no antihypertensive drug was almost 10%. All of these disappointing features, however, were more evident in men than in women and suggest their lower compliance to screening and treatment of hypertension. This finding should be taken into account by public health programs promoting the prevention of cardiovascular diseases at a community level.

Our findings are original, as no previous large epidemiological study has estimated the impact of hypertensive crises on hospital admissions using standardized procedures of data collection. Strength of our study relies on the numbers of recruiting centers, which were distributed all over the country under the universalistic coverage of the NHS and were representative of the Italian EDs. Moreover, standardized clinical procedures and data collection allowed us to provide unbiased results. Most important limitations of previous studies were the recruitment of cases from a single hospital, thus limiting the external validity of data, and the low numbers of examined people, thus limiting the power of results [13]–[15]. This paper is based on the analyses of more than 300,000 hospital admissions occurring over one year period in 10 EDs, allowing the identification of 1546 patients with hypertensive crises. Our data were not population-based and we could not estimate hospital admission rates for hypertensive crises over the study period. The study design, however, allowed us to increase the feasibility of a shared protocol among participating centers, thus providing data on the largest sample of patients with hypertensive crises examined up to now.

Our estimate of the impact of hypertensive crises on admissions to the EDs was similar to that provided by a single-center study conducted in Brazil [13], [15] and ten-fold lower than in another Italian single-center study performed 15 years ago [14]. The cross-sectional study design of previous Italian study and the present one does not allow to make inferences on a possible declining temporal trend of hypertensive crises in Italy. However, both studies used similar criteria and standardized methods of data collection, so that it is likely that a declining trend of hypertensive crises has occurred in Italy over time, as a result of both community screening programs and availability of new antihypertensive treatments.

Previous studies showed sex differences among patients admitted to the EDs for hypertensive crises, with a higher proportion of women than men [11]–[12], [15], [17]. In our study the numbers of admitted men and women were similar, but age was significantly lower in men. Moreover, the frequency of unknown hypertension was higher in men than in women. A larger proportion of men referred to take no anti-hypertensive drug, and the risk of having a hypertensive emergency was 34% higher in men than in women. All-together, these data suggest lower compliance to screening and antihypertensive treatment in men than in women and deserve future assessment, given its potential implication for public health programs.

Age of our patients was higher than in those examined by previous studies [13]–[15], reflecting the ageing of the general population, which is particularly relevant in Italy. Most of our patients were Europeans, as Africans and Asians represented 3% only of our patients, therefore our data cannot be extrapolated to populations with a larger proportion of ethnic groups at high risk for hypertensive crises [18]–[20].

With respect to hypertensive urgency, hypertensive emergency is a different clinical entity with high consumptions of resources, which in our study involved one over four patients. This proportion was very similar to that estimated in a previous Italian study [14]. More than 60% of our patients had either non-specific or non-cardiovascular presenting symptoms. Our multivariate analysis showed that patients with non-specific symptoms had 28% lower risk of having emergencies than urgencies, independently of age and sex. From a clinical point of view, however, the high frequency of non-specific presenting symptoms in both hypertensive emergencies and urgencies did not allow to perform differential diagnosis between these conditions without clinical monitoring and instrumental evaluation, which were required in the majority of our patients. Consistently with others, the most common signs/symptoms were dyspnea, chest pain, headaches and neurological deficit, which were related to the diagnosis of strokes, acute pulmonary edema and acute coronary syndromes [11], [15], [21].

In conclusion, this large Italian multicenter study provided evidence that hypertensive crises involved almost 5 out of 1,000 patients admitted over one year to the EDs and that hypertensive emergencies represented one fourth of all hypertensive crises. The high frequency of patients with unknown hypertension and the higher risk of emergencies in men than in women deserve future consideration, due to possible implications for public health programs.

## References

[1] Chobanian AV, Bakris GL, Black HR, Cushman WC, Green LA, et al (2003) The Seventh Report of the Joint National Committee on Prevention, Detection, Evaluation and Treatment of High Blood Pressure: the JNC 7 report. JAMA 289: 2560–2572. 12748199 10.1001/jama.289.19.2560

[2] Katz JN, Gore JM, Amin A, Anderson FA, Dasta JF, et al (2009) Practice patterns, outcomes, and end-organ dysfunction for patients with acute severe hypertension: the Studying the Treatment of Acute hyperTension (STAT) Registry. Am Heart J 158: 599–606. 19781420 10.1016/j.ahj.2009.07.020

[3] Mancia G, De Backer G, Dominiczak A, Cifkova R, Fagard R, et al (2007) Guidelines for the Management of Arterial Hypertension: The Task Force for the Management of Arterial Hypertension of the European Society of Hypertension (ESH) and of the European Society of Cardiology (ESC). J Hypertens 25: 1105–1187. 17563527 10.1097/HJH.0b013e3281fc975a

[4] Mancia G, De Backer G, Dominiczak A, Cifkova R, Fagard R, et al (2007) ESH-ESC Practice Guidelines for the Management of Arterial Hypertension ESH-ESC Task Force on the Management of Arterial Hypertension. J Hypertens 25: 1751–1762. 17762635 10.1097/HJH.0b013e3282f0580f

[5] Perez MI, Musini VM (2008) Pharmacological interventions for hypertensive emergencies. Cochrane Database Syst Rev. 2008 Jan 23 (1): CD003653. 10.1002/14651858.CD003653.pub3 PMC6991936 18254026

[6] Marik PE, Varon J (2007) Hypertensive crisis. Challanges and Management. Chest 131: 1949–1962. 17565029 10.1378/chest.06-2490

[7] Webster J, Petrie JC, Jeffers TA, Lovell HJ (1993) Accelerated hypertension – patterns of mortality and clinical factors affecting outcome in treated patients. Q J Med 86: 485–493. 8210306 10.1093/qjmed/86.8.485

[8] National High Blood Pressure Education Program (2004) The seventh report of the Joint National Committee on prevention, detection, evaluation and treatment of high blood pressure. Bethesda (MD): Dept. Of Health and Human Services, National Institutes of Health, National Heart, Lung and Blood Institute; 2004. NIH Publication No.04–5230. 20821851

[9] Vlcek M, Bur A, Woisetschager C, Herkner H, Laggner AN, et al (2008) Association between hypertensive urgencies and subsequent cardiovascular events in patients with hypertension. J Hypertens 26: 657–662. 18327073 10.1097/HJH.0b013e3282f4e8b6

[10] Cherney D, Straus S (2002) Management of Patients With Hypertensive Urgencies and Emergencies. J Gen Intern Med 17: 937–945. 12472930 10.1046/j.1525-1497.2002.20389.x PMC1495142

[11] Elijovich F, Laffer CL (2010) Acute Stroke. Lower Blood Pressure Looks Better and Better. Hypertension 56: 808–810. 20823376 10.1161/HYPERTENSIONAHA.110.159038

[12] Slama M, Modeliara SS (2006) Hypertension in the intensive care unit. Curr Opin Cardiol 21: 279–287. 16755195 10.1097/01.hco.0000231396.56738.d8

[13] Vilela Martin JF, Higashiama E, Garcia E, Luizon MR, Cipullo JP (2004) Hypertensive crisis profile. Prevalence and clinical presentation. Arquivos Brasileiro de Cardiologia 83: 131–136. 10.1590/s0066-782x2004001400004 15322655

[14] Zampaglione B, Pascale C, Marchisio M, Cavallo Perin P (1996) Hypertensive urgencies and emergencies. Prevalence and clinical presentation. Hypertension 27: 144–147. 8591878 10.1161/01.hyp.27.1.144

[15] Vilela Martin JF, Oliveira Vaz-de-Meolo R, Hiromi Kuniyoshi C, Abdo AN, Yugar-Toledo JC (2010) Hypertensive crisis. Clinical-epidemiological profile. Hypertension Research 1: 1–5. 10.1038/hr.2010.245 21160483

[16] Levey AS, Coresh J, Greene T, Stevens LA, Zhang YL, et al (2006) Using standardized serum creatinine values in the modification of diet in renal disease study equation for estimating glomerular filtration rate. Ann Intern Med 145: 247–254. 16908915 10.7326/0003-4819-145-4-200608150-00004

[17] Cerrilo MR, Hernandez PM, Pinilla CF, Martell Claros N, Luque Otero M (2002) Hypertensive crises: prevalence and clinical aspects. Rev Clin Esp 202: 255–258. 12060538 10.1016/s0014-2565(02)71046-1

[18] Hua D, Yub D (2010) Epidemiology of cardiovascular disease in Asian women. Nutrition, Metabolism Cardiovascular Diseases 20: 394–404. 10.1016/j.numecd.2010.02.016 20591635

[19] Liu SL, Caguioa ES, Park CG (2006) Reducing stroke risk in hypertensive patients: Asian Consensus Conference Recommendations. International Journal of Stroke 1: 150–157. 18706035 10.1111/j.1747-4949.2006.00041.x

[20] World Health Organization Statistical Information System (WHOSIS) (2004) Estimates by WHO Region. Mortality Geneva: World Health Organization, 2004 (www document). Available: http://www.who.int/healthinfo/global_burden_disease/2004_report_update/en/ Accessed 2014 Mar 10.

[21] Saguner AM, Dur S, Perring M, Schiemann U, Stuck AE, et al (2010) Risk factors promoting hypertensive crisis: evidence from a longitudinal study. Am J Hypertens 23: 775–780. 20395943 10.1038/ajh.2010.71

